# Effects of summer flooding on hormones and metabolic enzymes in *Myricaria laxiflora* during recovery growth

**DOI:** 10.1093/conphys/coaf078

**Published:** 2025-12-15

**Authors:** Yangyun Liu, Ning Wang, Liu Sun, Zhuodan Han, Yongwen Huang, Fangqing Chen

**Affiliations:** Hubei International Scientific and Technological Center of Ecological Conservation and Management in the Three Gorges Area, China Three Gorges University, Yichang, 443002 Hubei Province, PR China; Hubei International Scientific and Technological Center of Ecological Conservation and Management in the Three Gorges Area, China Three Gorges University, Yichang, 443002 Hubei Province, PR China; Hubei International Scientific and Technological Center of Ecological Conservation and Management in the Three Gorges Area, China Three Gorges University, Yichang, 443002 Hubei Province, PR China; Hubei International Scientific and Technological Center of Ecological Conservation and Management in the Three Gorges Area, China Three Gorges University, Yichang, 443002 Hubei Province, PR China; Hubei International Scientific and Technological Center of Ecological Conservation and Management in the Three Gorges Area, China Three Gorges University, Yichang, 443002 Hubei Province, PR China; Engineering Research Center of Eco-environment in Three Gorges Reservoir Region, Ministry of Education, China Three Gorges University, Yichang, 443002 Hubei Province , PR China

**Keywords:** Flooding, hormone, metabolic enzyme, *Myricaria laxiflora*, recovery growth

## Abstract

Remnant populations of *Myricaria laxiflora* on river islands along the Yangtze River enter dormancy and endure varying degrees of flooding in summer, with their growth and development recovering in autumn. In this study, *M*. *laxiflora* plants were subjected to controlled flooding, and the changes in plant hormones and metabolic enzymes in different stages of recovery growth were measured to elucidate the biochemical mechanisms of summer flooding on plant recovery. Our findings indicated that flooding duration and depth significantly affected the levels of hormones during recovery growth. Compared to the control, cytokinin (CTK), gibberellin (GA) and abscisic acid (ABA) increased by 120.04%–178.53%, 26.07%–56.20% and 36.71%–79.81, respectively, while indole-3-acetic acid (IAA) decreased by 4.88%–26.38% with different flooding durations. Moreover, summer flooding altered metabolic enzymes in *M*. *laxiflora* during recovery growth. Under different flooding durations, ribulose-1,5-bisphosphate carboxylase/oxygenase (RuBisCO) and RuBisCO-activating enzyme (RCA) increased by 117.94%–185.93% and 55.51%–98.19%, respectively. With different flooding depths, RCA increased by 107.12%–190.55%, while phosphoenolpyruvate carboxylase (PEPC) decreased by 9.37%–20.92%. Pearson’s correlation analysis indicated relationships between the changes in hormones (IAA, ABA, CTK and GA) and enzymes (RCA, RuBisCO and PEPC) induced by summer flooding. These correlations indicated that the alternations of hormones induced by summer flooding may influence plant physiology through the modulation of metabolic enzymes. The increasing CTK, GA, ABA, RuBisCO and RCA, and decreasing IAA and PEPC would enhance photosynthetic physiology and mitigate respiratory physiology, thereby facilitating plant recovery growth. It is suggested that riverbanks for population restoration of *M*. *laxiflora* have to annually experience a period of flooding in the *in situ* conservation.

## Introduction

Fluctuations in water levels represent an important ecological factor influencing the growth and development of riparian vegetation undergoing flooding stress in summer. Flooding stress usually affects the expression of genes related to specific endogenous hormones and metabolic enzymes pivotal to plant growth, leading to changes in hormone and enzyme content in plants (Voesenek and Bailey-Serres, 2015； Wurms *et al.*, 2023). Concentrations of endogenous hormones that facilitate the formation and growth of branches and leaves, including gibberellin (GA), indole-3-acetic acid (IAA) and cytokinin (CTK), are significantly reduced (Kim *et al.*, 2015). Conversely, hormones that inhibit branch and leaf growth and facilitate their abscission, such as abscisic acid (ABA) and ethylene, are markedly elevated (Loreti *et al.*, 2016; González-Guzmán *et al.*, 2021; Cisse *et al.*, 2022). Enzymes associated with photosynthesis, such as ribulose-1,5-bisphosphate carboxylase/oxygenase (RuBisCO) and RuBisCO-activating enzyme (RCA), typically exhibit reduced levels, whereas enzymes related to respiration, such as phosphoenolpyruvate carboxylase (PEPC), tend to increase (Komatsu *et al.*, 2014). These alternations lead to a considerable decrease in plant physiological activity (Irfan *et al.*, 2010). Although many plants are capable of recovery from flooding stress, their overall growth and development are substantially impaired (Vartapetian and Jackson, 1997). However, some species have adapted to flooding stress through summer dormancy (Chen and Xie, 2009a; Zhou *et al.*, 2021). Despite experiencing similar changes in metabolic enzymes and endogenous hormones as non-adapted plants during flooding (Benschop *et al.*, 2005), these plants exhibit enhanced branch and leaf formation and growth during recovery as compared to plants not experiencing flooding stress (Li *et al.*, 2013). Studies have shown that flooding can enhance the photosynthetic efficiency of these plants during recovery growth (Guan *et al.*, 2020a). However, the biochemical mechanisms underlying their adaptation are not fully understood.

*Myricaria laxiflora* in the Tamaricaceae family is a shrub distributed along the banks of the Yangtze River from Zhijiang City in Hubei Province to Wanzhou City in Sichuan Province of China. The construction of the Three Gorges Dam and Gezhouba Dam has resulted in the destruction of the primary habits of *M*. *laxiflora* within the Three Gorges Reservoir region, consequently endangering this species. Currently, only remnant populations exist on several river islands along the Yangtze River downstream of the Three Gorges Dam and Xiangjiaba Dam (Huang *et al.*, 2023). Between June and September, the habitats of these populations experience summer flooding for different periods, and post-flooding recovery growth has been observed (Chen and Xie, 2009b; Chen and Wang, 2015). In recent studies, we found that the branches and leaves of this species turned yellow and withered in early May, indicating the onset of dormancy (Chen and Xie, 2009a, 2009b), which was triggered by increasing daylight hours (Zhou *et al.*, 2020). This dormancy persists throughout the summer flooding period (Chen and Xie, 2009a, 2009b; Zhou *et al.*, 2021). The growth and sexual reproduction of *M*. *laxiflora* resumed after flooding, but the role of summer flooding in releasing dormancy remains to be further investigated. Evidence shows enhanced photosynthetic efficiency and leaf and branch growth in *M*. *laxiflora* individuals subjected to summer floods, in contrast to those not exposed to flooding (Guan *et al.*, 2020a, 2020b). Consequently, we hypothesized that flooding during summer dormancy may facilitate the recovery growth of *M*. *laxiflora*. To test this hypothesis, plants were subjected to varying levels of flooding during summer dormancy, and the content of endogenous hormones and critical metabolic enzymes was measured at different recovery stages. Through comparative analysis of treatment outcomes, this research aims to elucidate the impacts and underlying biochemical mechanisms of flooding during summer dormancy on plant growth during the recovery period.

## Materials and methods

### Experimental design

An experimental design for two variables at three levels was established. The levels of flooding depth were root-flooding (water level at the soil surface), semi-flooding (water level at 5 cm above the soil surface) and fully flooding (water level at 10 cm above the plant top). The levels of flooding duration were 10, 50 and 90 days. The combination of these two variables at three levels resulted in a set of nine experiments. Additionally, a control (CK) without flooding was added, totalling 10 treatments. Each treatment was performed in 18 replicates, and a total of 180 experimental units were included. Two-year-old seedlings of *M. laxiflora* exhibiting uniform growth were individually planted in pots. For plants in the nine combinations of flooding depth and duration, treatments with three flooding depths started on 16 June, 25 July and 5 September 2022, respectively. Water in the flooded pots was refreshed every three days. Plants in CK were watered every three days. On 15 September 2022, excess water was removed and all plants were watered every three days during the following recovery growth. Three plants were randomly selected from each treatment at the early (day 10), middle (day 50) and late (day 90) stages of recovery growth. The newly grown secondary branches and leaves in the middle and upper parts of the plants were collected and placed in labelled centrifuge tubes. These samples were kept in liquid nitrogen for 12 h and then stored at −80°C. Thirty samples (3 replicates × 10 treatments) were obtained at each stage, and a total of 90 samples were collected.

### Determination of hormones during recovery growth

Indirect enzyme-linked immunosorbent assay (ELISA, Wuhan Punes Biotechnology Co., Ltd, China) was used to determine the content of CTK, GA, IAA and ABA (Wu *et al.*, 1988). Precisely 0.1 g of fresh sample was placed in a pre-cooled mortar with extraction buffer and thoroughly ground. The homogenate was centrifuged at 4°C and 1000 ×*g* for 20 min. The supernatant was used as the hormone extract. This extract, along with standards and horseradish peroxidase-labelled antibodies, was sequentially added to an ELISA microplate (Meimian, China) coated with antibodies against IAA, ABA, CTK and GA. The microplate was incubated at 37°C for 60 min and washed 3–4 times with Tween 20-containing buffer to remove unbound antigens and secondary antibodies and non-specifically adsorbed substances. Colour development was performed using 3,3′,5,5′-tetramethylbenzidine as the substrate. Absorbance was measured at 450 nm with a microplate reader (Sunrise, Switzerland) and used to calculate the content of CTK, GA, IAA and ABA.

### Determination of metabolic enzymes during recovery growth

Indirect ELISA was used to determine the content of RuBisCO, RCA and PEPC closely related to plant photosynthetic physiology and respiration (Wu, 1988). Precisely 0.1 g of fresh sample was placed in a pre-cooled mortar, combined with extraction buffer, and thoroughly ground. The homogenate was centrifuged at 4°C and 1000× *g* for 20 min, and the supernatant was used as the enzyme extract. This extract, together with standards and horseradish peroxidase-labelled antibodies, was sequentially added to an ELISA microplate (Wuhan Punes Biotechnology Co., Ltd, China) coated with antibodies against RCA, RuBisCO and PEPC. The microplate was incubated at 37°C for 60 min and washed thoroughly. Colour development was performed using 3,3',5,5'-tetramethylbenzidine as the substrate. Absorbance was measured at 450 nm with a microplate reader (Sunrise, Switzerland) and used to calculate the content of RuBisCO, RCA and PEPC.

### Data analysis

The content of hormones (IAA, ABA, CTK, GA) and enzymes (RuBisCO, RCA, PEPC) were set as dependent variables, and the flooding depth and duration were set as independent variables, to perform multivariate analysis of variance. Multiple comparisons were performed using the least significant difference (LSD) test to evaluate the differences between treatments and the differences between recovery growth stages in the same treatment. Pearson’s correlation coefficient was used to examine the correlations between hormones and enzymes during recovery growth to elucidate the mechanisms by which summer flooding influences plant physiological and biochemical processes during recovery growth. Data were analyzed using SPSS 26.0 (IBM Corp., Armonk, USA) and plotted with OriginPro 2022 (OriginLab, Northampton, USA).

## Results 

### Effects of flooding on hormones during the recovery growth of *M. laxiflora*

The flooding duration significantly affected the CTK, IAA and ABA content during the recovery growth (*P* = 0.000; *P* = 0.000; *P* = 0.000), so did the flooding depth (*P* = 0.006; *P* = 0.000; *P* = 0.000). The flooding duration also had a significant effect on GA content (*P* = 0.001), however, the effect of flooding depth on GA content was not significant (*P* = 0.083). The interactions between flooding duration and flooding depth had no significant effect on the content of CTK, IAA, ABA and GA (*P* = 0.893; *P* = 0.997; *P* = 0.948; *P* = 0.996).

**Figure 1:**
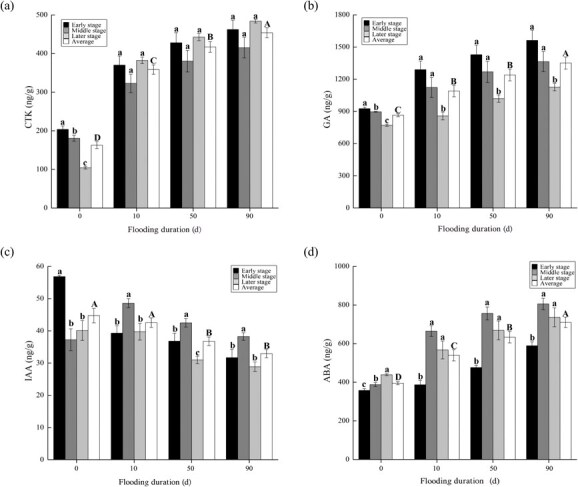
Content (mean ± standard error) of cytokinin (**a**), gibberellin (**b**), indole-3-acetic acid (**c**) and abscisic acid (**d**) in plants during recovery growth from treatments with different flooding durations. Capital letters indicate differences between treatments throughout the recovery growth (*P* < 0.05), and lowercase letters indicate differences between stages of recovery growth in the same treatment (*P* < 0.05).

The levels of CTK, GA and ABA increased with increasing flooding duration (Fig. 1). Compared to CK, CTK, GA and ABA increased by 120.04%–178.53%, 26.07%–56.20% and 36.71%–79.81%, respectively. However, IAA decreased with increasing flooding duration, with decreases of 4.88%–26.38% relative to CK. The effect of flooding duration on plant hormone content varied with plant recovery growth. GA of the flooded plants significantly decreased and IAA and ABA increased and then decreased, while CTK decreased and then increased.

**Figure 2:**
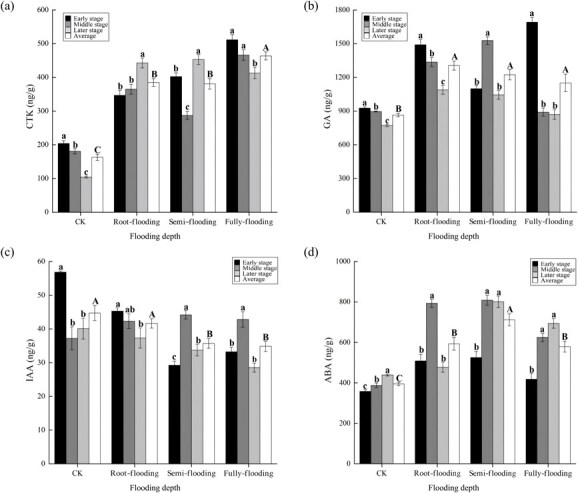
Content (mean ± standard error) of cytokinin (**a**), gibberellin (**b**), indole-3-acetic acid (**c**) and abscisic acid (**d**) in plants during recovery growth from treatments with different flooding depths. Capital letters indicate differences between treatments throughout the recovery growth (*P* < 0.05), and lowercase letters indicate differences between stages of recovery growth in the same treatment (*P* < 0.05).

In plants treated with different flooding depths in summer, the levels of CTK, ABA and GA significantly increased, while IAA significantly decreased in flooded plants compared to CK during recovery growth (Fig. 2). Specifically, CTK, GA and ABA showed increases of 133.71%–184.57%, 33.09%–50.95% and 46.56%–80.19%, respectively, while IAA exhibited a decrease of 6.89%–22.06%. With increasing flooding depth, CTK increased, ABA increased and then decreased, and GA and IAA decreased. The effect of flooding depth on plant hormone content varied with plant recovery growth. The CTK levels significantly increased in root-flooded plants, decreased and then increased in semi-flooded plants, and significantly decreased in fully-flooded plants. The levels of GA significantly decreased in root- and fully-flooded plants but increased and then decreased in semi-flooded plants, whereas IAA significantly decreased in root-flooded plants but increased and then decreased in semi- and fully flooded plants. The ABA levels increased and then decreased in root- and semi-flooded plants but significantly increased in fully flooded plants.

### Effects of flooding on metabolic enzymes during the recovery growth of *M. laxiflora*

The flooding duration significantly influenced the level of RCA, Rubisco during the recovery growth (*P* = 0.000; *P* = 0.000), but did not the level of PEPC (*P* = 0.842). The flooding depth significantly affected the level of RCA and PEPC (*P* = 0.000; *P* = 0.016), but but did not the level of Rubisco (*P* = 0.846). The interaction between flooding time and flooding depth had a significant effect on the level of PEPC (*P* = 0.000), but had no significant effects on the level of RCA and Rubisco (*P* = 0.986; *P* = 0.999).

**Figure 3:**
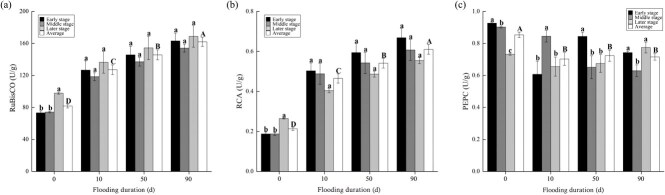
Content (mean ± standard error) of ribulose-1,5-bisphosphate carboxylase/oxygenase (**a**), ribulose-1,5-bisphosphate carboxylase/oxygenase-activating enzyme (**b**) and phosphoenolpyruvate carboxylase (**c**) in plants during recovery growth from treatments with different flooding durations. Capital letters indicate differences between treatments throughout the recovery growth (*P* < 0.05), and lowercase letters indicate differences between stages of recovery growth in the same treatment (*P* < 0.05).

Compared to CK, RuBisCO and RCA were significantly higher in flooded plants and their levels increased with increasing flooding duration (Fig. 3). Specifically, RCA and RuBisCO increased by 117.94%–185.93% and 55.51%–98.19%, respectively, relative to CK. Conversely, PEPC did not show significant changes in plants treated with different flooding durations, but significantly decreased by 15.26%–17.70% relative to CK, and its level decreased with increasing flooding duration. The effects of flooding duration on metabolic enzymes varied considerably with recovery growth. In flooded plants, RCA decreased and RuBisCO decreased and then increased, whereas PEPC increased and then decreased in plants treated with 10 days of flooding, but decreased and then increased in plants treated with 50- and 90-day flooding durations.

**Figure 4:**
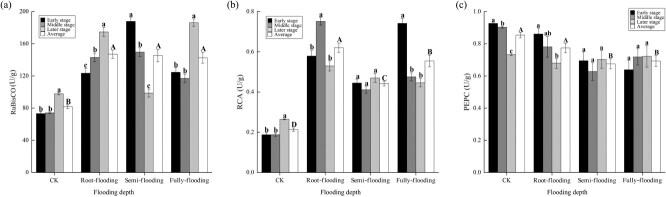
Content (mean ± standard error) of ribulose-1,5-bisphosphate carboxylase/oxygenase (**a**), ribulose-1,5-bisphosphate carboxylase/oxygenase-activating enzyme (**b**) and phosphoenolpyruvate carboxylase (**c**) in plants during recovery growth from treatments with different flooding depths. Capital letters indicate differences between treatments throughout the recovery growth (*P* < 0.05), and lowercase letters indicate differences between stages of recovery growth in the same treatment (*P* < 0.05).

Under the influence of flooding depth, the level of RCA significantly increased during recovery growth, while PEPC significantly decreased (Fig. 4). Specifically, RCA increased by 107.12%–190.55%, while PEPC decreased by 9.37%–20.92% compared to CK. RuBisCO did not show significant variations at different flooding depths but significantly increased by 74.38%–79.82% relative to CK. The effects of flooding depth on plant metabolic enzymes varied considerably with recovery growth. The levels of RCA increased and then decreased in root-flooded plants, decreased and then increased in semi-flooded plants, and decreased in fully flooded plants. By contrast, RuBisCO significantly increased in root-flooded plants, significantly decreased in semi-flooded plants, and decreased and then increased in fully flooded plants. The levels of PEPC significantly decreased in root-flooded plants, decreased and then increased in semi-flooded plants, and significantly increased in fully flooded plants.

### Correlations between hormones and metabolic enzymes

During recovery growth, correlations were identified between hormones and between enzymes (Table 1). In terms of hormones, IAA was significantly negatively correlated with ABA and CTK, while ABA was significantly positively correlated with CTK and GA but significantly negatively correlated with IAA. For CTK, significant positive correlations were observed with ABA and GA, while significant negative correlations were observed with IAA. For GA, significant positive correlations were detected with ABA and CTK. In terms of enzymes, RCA was significantly positively correlated with RuBisCO but significantly negatively with PEPC. There was a significant negative correlation between RuBisCO and PEPC.

During recovery growth, correlations were also identified between hormones and enzymes (Table 1). We found that CTK was positively correlated with RCA and RuBisCO but negatively with PEPC; GA was positively correlated with RCA and RuBisCO; IAA was positively correlated with PEPC but negatively with RCA and RuBisCO; and ABA was positively correlated with RCA and RuBisCO but negatively with PEPC.

**Table 1:** Pearson’s correlation analysis of changes in the content of hormones and metabolic enzymes during recovery growth

	IAA	ABA	CTK	GA	RCA	Rubisco	PEPC
IAA	1.000						
ABA	−0.290^**^	1.000					
CTK	−0.475^**^	0.414^**^	1.000				
GA	−0.088	0.274^**^	0.434^**^	1.000			
RCA	−0.330^**^	0.444^**^	0.759^**^	0.717^**^	1.000		
Rubisco	−0.539^**^	0.435^**^	0.555^**^	0.288^**^	0.515^**^	1.000	
PEPC	0.247^**^	−0.213^*^	−0.298^**^	−0.187	−0.245^**^	−0.329^**^	1.000

Note: ^*^  *P* ≤ 0.05; ^**^  *P* ≤ 0.01

## Discussion

### Effects of summer flooding on hormones during recovery growth

*M. laxiflora* enters deep dormancy during prolonged flooding in summer to adapt to the stress caused by high temperatures and drought following the flooding of riverbanks (Chen *et al.*, 2019). Our earlier research indicated that the growth and photosynthetic physiology of *M*. *laxiflora* were improved in plants exhibiting summer dormancy (Chen and Wang, 2015), suggesting that summer flooding might facilitate plant recovery growth. This observation was confirmed in the current investigation into the impact of summer flooding on plant hormones during recovery growth. We showed that CTK, GA and ABA were significantly higher, while IAA was lower or significantly lower, in flooded plants compared to CK. With the increase in flooding duration, CTK, GA and ABA increased, while IAA decreased. CTK is known to interrupt plant dormancy and promote bud differentiation and plant growth (Hwang *et al.*, 2012). Additionally, GA plays a crucial role in cell elongation and organ growth (Sponsel, 2018). Consequently, flooding-induced increases in CTK and GA could encourage dormancy release and vegetation growth. Furthermore, ABA enhances plant resistance to stresses (Boudsocq *et al.*, 2004; Kuromori *et al.*, 2018), while IAA induces tissue differentiation and floral organ formation and reduces apical dominance (Friml, 2003). The increase in ABA and decrease in IAA indicate that flooding not only enhanced stress resistance during recovery growth but also promoted plant lateral branch growth and sexual reproduction.

Endogenous hormones exhibit distinct change patterns during the release of plant dormancy. Specifically, GA, CTK and IAA, which promote cell division and expansion and the formation and growth of branches and leaves, tend to increase. Conversely, ABA and ethylene, which facilitates cell senescence and apoptosis as well as dormancy and abscission of the branches and leaves, shows a decrease (Zheng *et al.*, 2009; Liu *et al.*, 2013; Li *et al.*, 2020). In the present study, however, GA, CTK, IAA and ABA showed change patterns during recovery growth that differ from previous investigations, potentially reflecting the unique growth and development dynamics of this species. The branches of *M*. *laxiflora* germinated and grew immediately after the termination of flooding, which was followed by flowering and fruiting and then vegetative growth (Chen *et al.*, 2019; Sun *et al.*, 2024). In the early stage of recovery growth, the flooding-induced increases in CTK- and GA-facilitated vegetative growth. Their levels decreased during reproductive growth and then increased during vegetative growth. Both IAA and ABA increased and then decreased during recovery growth. The increases in IAA and ABA in the middle stage of recovery contributed to stress resistance and sexual reproduction. Due to limitations in experimental conditions, this study did not measure the changes in ethylene. However, related research indicated that ethylene level in plant tissues typically increased during dormancy and decreased during the recovery growth (Larsen *et al.*, 1986; Banga *et al.*, 2010).

### Effects of summer flooding on metabolic enzymes during recovery growth

Plant photosynthesis and respiration are regulated by a variety of enzymes. RuBisCO catalyses the carboxylation and oxygenation reactions of ribulose 1,5-bisphosphate, thereby facilitating the fixation of atmospheric CO_2_ in plant cells (Andersson and Backlund, 2008). RuBisCO-activating enzyme activates RuBisCO and enhances its carboxylase or oxygenase activity and thus photosynthetic efficiency (He *et al.*, 2017), whereas PEPC plays an important role in non-photosynthesis processes, particularly in the anaerobic respiration pathway, by replenishing intermediates in the tricarboxylic acid cycle, participating in the regulation of chloroplast metabolism, and directing the flux of carbon into the respiratory pathway (Champigny and Foyer, 1992; Smith *et al.*, 1992; Oaks, 1994; O’Leary *et al.*, 2011). The vegetative growth of *M*. *laxiflora* recovered immediately after the termination of flooding (Chen *et al.*, 2019). Our previous research showed that branch and leaf growth and photosynthetic physiology were relatively high in flooded plants (Chen and Xie, 2009a; Guan *et al.*, 2020a, 2020b). In the current study, RCA and RuBisCO were significantly elevated during recovery growth in flooded plants than in CK, whereas PEPC was notably reduced. With the increase in flooding duration, RCA and RuBisCO increased, while PEPC decreased significantly. Similarly, RCA and RuBisCO increased, while PEPEC decreased in treatments with different flooding depths, with different enzymes showing various change patterns with increasing flooding depth. This result suggests that summer flooding not only promoted photosynthetic efficiency but also attenuated respiratory intensity to support plant growth and development during recovery.

Alterations in plant metabolic enzyme activity correspond to changes in photosynthetic physiology and respiration (Moore *et al.*, 2021). After flooding, the photosynthetic physiology of the remnant populations of *M*. *laxiflora* was high due to compensatory growth in the early stage of recovery, following which it decreased in the middle stage due to drought caused by the decrease in the water level in the environment, and then increased (Guan *et al.*, 2020a, 2020b). In the present study, RuBisCO decreased and then increased during recovery growth in the flooded plants, RCA decreased and PEPC in plants subjected to 50- and 90-day flooding decreased and then increased. These patterns are consistent with the changes in photosynthetic physiology after flooding (Chen and Xie, 2009a, 2009b).

### Correlations between hormones and metabolic enzymes during recovery growth

Plant hormones play crucial roles not only in directly influencing cell division and growth as well as organ differentiation and growth, but also in impacting plant photosynthetic physiology and respiration through the modulation of metabolic enzyme activities (Müller and Munné-Bosch, 2021). Studies have demonstrated that CTK significantly increased the instantaneous apparent photosynthetic rate, chlorophyll content, overall chloroplast electron transport rate and RuBisCO activity in tobacco leaves, thereby prolonging their photosynthetic functionality (Jiang *et al.*, 2006). Gibberellic acid regulates photosynthesis through 37 photosynthetically differentially expressed genes including GID1, RGA, GID2 and MYBGa, resulting in a significant increase in aboveground plant biomass (Xie *et al.*, 2016). Indole-3-acetic acid is known to elevate the content of plant photosynthetic pigments and the activities of photosynthetic enzymes (Singh and Prasad, 2015), whereas ABA promotes chlorophyll degradation and diminishes leaf photosynthetic rate, carbonic anhydrase activity, RuBisCO activity and the electron transfer capacity of photosystems I and II (Wei and Ning , 2002). In the present study, CTK and GA in the flooded plants were positively correlated with RuBisCO and RCA, and CTK was negatively correlated with PEPC. This indicates that changes in CTK and GA induced by summer flooding could promote photosynthetic physiology and reduce respiration during recovery growth through the modulation of metabolic enzymes. Conversely, IAA was positively correlated with PEPC and negatively correlated with RuBisCO and RCA, whereas ABA was positively correlated with RuBisCO and RCA and negatively correlated with PEPC. These findings diverge from previous research and might be related to post-flooding sexual reproduction and vigorous growth that requires relatively high stress resistance. The decrease in IAA inhibited apical dominance and promoted sexual reproduction, whereas the increase in ABA enhanced plant resistance required by vigorous recovery growth (Zhang *et al.*, 2012).

## Conclusions

Populations of *M*. *laxiflora* undergo various degrees of flooding during summer dormancy. Our previous research revealed that flooding enhanced photosynthetic physiology and branch and leaf growth during recovery in autumn. In the present study, controlled flooding was performed to determine the changes in key hormones and metabolic enzymes during the recovery growth of plants subjected to different levels of flooding, with the aims of further elucidating the biochemical mechanisms by which summer flooding promotes plant recovery growth. Our results showed that flooding duration had highly significant impacts on CTK, GA, IAA and ABA during recovery growth. Specifically, CTK, GA and ABA were significantly higher in plants treated with different flooding durations when compared to CK, while IAA was significantly reduced. Additionally, flooding duration significantly affected RuBisCO and RCA, which were significantly increased in plants under different flooding durations. Although PEPC showed non-significant differences between various flooding durations, PEPC was significantly lower in flooded plants than in CK plants. Flooding depth significantly affected IAA, ABA, CTK as well as RCA and PEPC in *M*. *laxiflora* plants during recovery growth. We found that CTK, GA and ABA were significantly higher, whereas IAA was significantly lower in plants treated with different flooding depths when compared to CK. An increase in RCA but a decrease in PEPC was detected in plants treated with different flooding depths. Pearson’s correlation analysis revealed that the flooding-induced changes in CTK, GA and ABA were positively correlated with RuBisCO and RCA, and CTK and ABA were negatively correlated with PEPC. Additionally, IAA was negatively correlated with RuBisCO and RCA but positively correlated with PEPC. Therefore, the increasing CTK, GA, ABA, RuBisCO, RCA and decreasing IAA, PEPC induced by summer flooding would enhance photosynthetic physiology and mitigate respiratory physiology, thereby facilitating plant recovery growth. It is suggested that riverbanks for population restoration of *M*. *laxiflora* have to annually experience a period of flooding in the *in situ* conservation.

## Data Availability

Data are available upon request from the corresponding author.
